# Qualitative interviews in patients with lipodystrophy to assess the patient experience: evaluation of hunger and other symptoms

**DOI:** 10.1186/s41687-022-00486-3

**Published:** 2022-07-29

**Authors:** Susan A. Martin, Robert J. Sanchez, Oyebimpe Olayinka-Amao, Charles Harris, Sheri Fehnel

**Affiliations:** 1 RTI Health Solutions, Research Triangle Park, NC USA; 2 Regeneron Pharmaceuticals, Inc., Tarrytown, NY USA; 3 RTI Health Solutions, 3005 Boardwalk Street, Suite 105, Ann Arbor, MI 48105 USA

**Keywords:** Hunger, Leptin, Generalized lipodystrophy, Partial lipodystrophy, Patient-reported outcomes

## Abstract

**Background:**

New treatments are being evaluated for lipodystrophy; however, limited information is available on the patient experience. Results of a prior patient panel showed that hunger and temperature-related symptoms were an issue for participants. Therefore, evaluation of any changes in these symptoms is recommended for inclusion in new treatment options. The objective of this study was to further understand the patient experience and to evaluate newly developed items of hunger and temperature regulation.

**Methods:**

Individual, in-depth telephone interviews were conducted via semi-structured discussion guide. Telephone interviews were conducted with 21 US patients with generalized lipodystrophy (GLD) or partial lipodystrophy (PLD). Eligibility requirements included self-reported PLD or GLD. Interviews included open-ended concept elicitation followed by a review of newly developed items assessing hunger, temperature sensations, and patient globals. Interviews were conducted in two rounds, with the newly developed items assessing hunger revised after each round of interviews based on participant feedback.

**Results:**

Results indicated that hunger-related symptoms were considered a current issue for greater than half (N = 11) of participants, and all but one reported this as an issue at some point in their lives. Specifically, participants most often reported symptoms of increased appetite and not feeling full. The cognitive debriefing process indicated that the hunger-related symptoms, temperature, and global impression of change and severity items were correctly interpreted and easily completed by the participants. While not a focus of the interviews, the concept elicitation results demonstrated that pain was a frequently reported and bothersome symptom in this patient population.

**Conclusions:**

This qualitative research provided evidence to support the use of clinical outcomes assessments such as hunger and temperature-related items in clinical trials.

**Supplementary Information:**

The online version contains supplementary material available at 10.1186/s41687-022-00486-3.

## Introduction

Adipose tissue is recognized as an endocrine organ, with adipocytes, the primary cells in adipose tissue, required for control of homeostatis via the secretion of hormones or adipokines such as leptin [1, 2]. Circulating leptin levels are proportional to an individual’s mass of adipose tissue, and serve as a signal to the brain reflecting energy stores [3]. Lipodystrophy is a term used to describe various rare conditions characterized by a generalized or partial loss of adipose tissue [4–6]. Patients with generalized lipodystrophy (GLD) are severely deficient in leptin due to the near absence of adipose tissue. Similarly, patients with partial lipodystrophy (PLD) have decreased leptin levels, but to a lesser degree than patients with GLD. Decreased leptin levels lead to increased hunger in patients with GLD and some patients with PLD. Leptin replacement with metrelepin in patients with lipodystrophy results in increased satiety [7]. In addition, behavioral tests demonstrated that perceived hunger, importance of eating, eating frequencies, and liking ratings of food pictures significantly decreased during metreleptin therapy in patients with lipodystrophy [7].

Both GLD and PLD can be due to gene mutations (congenital generalized lipodystrophy [CGL] and familial PLD [FPL], respectively) [4, 5]. CGL is due to mutations in any one of four genes, designated CGL1-4, whereas FPL can be due to mutations in any one of six or more genes, designated FPLD2-7. FPLD1, or Kobberling’s syndrome, is not a monogenic disorder but has a strong familial component. In addition, both GLD and PLD can be acquired, resulting from other conditions such as autoimmune diseases characterized by immune destruction of leptin-producing adipocytes [4, 5, 8].

Patients with lipodystrophy suffer from metabolic abnormalities, including diabetes, hypertriglyceridemia, and fatty liver [8–10]. In extreme cases, these abnormalities can result in severe symptoms, for example, pancreatitis. However, more recently we are beginning to understand that patients with lipodystrophy are suffering on a daily level with conditions that are more difficult to quantify when compared with laboratory measures such as glucose and triglycerides. Due to the rarity of lipodystrophies, primary qualitative research to understand the patient experience is limited.

To inform symptom assessment for the new treatments being studied, an informal patient advisory meeting with seven individuals with PLD or GLD and/or their caregivers was conducted in 2018. Issues with hunger related to lipodystrophy were reported by all of the participants. Additional symptoms reported included fatigue, difficulty in temperature regulation, and pain. Based on these patient inputs, patient-reported outcome (PRO) items were developed to assess hunger-related symptoms and temperature regulation for inclusion in upcoming clinical trials.

PRO measures provide a standardized approach to assess the impact of a disease from the patient perspective by evaluating symptoms, functioning, and health-related quality of life. In addition, PROs complement the clinical endpoints typically assessed in clinical trials [11]. Many major regulatory authorities consider PRO data an important means of capturing the patient perspective during the drug development process; therefore, guidance on the evaluation of PRO measures proposed for label claims and papers on the use of PRO measures are available [12, 13]. A key component detailed in these guidance documents is the need for input from the specific target population. A review of the literature on patients with lipodystrophy for available instruments did not identify any measures for hunger and temperature regulation that were developed based on input from the target patient population [4, 6–10], hence the need for the new PRO items following the initial patient advisory meeting described above.

The objective of the current study was to further evaluate the patient experience of hunger-related symptoms and other relevant patient symptoms in individuals with lipodystrophy, and to evaluate the newly developed items for comprehension, ease of completion, and content validity.

## Methods

This study followed the recommendations of the US Food and Drug Administration, as described in the agency’s guidance on PROs, “Patient-Reported Outcomes Measure: Use in Medicine Product Development to Support Labelling Claims” [12]. The study was reviewed and approved by RTI International’s institutional review board. Informed consent was obtained from each participant prior to participation.

### Patient recruitment

All patients were recruited via collaboration with Lipodystrophy United, a patient advocacy organization in the USA. Lipodystrophy United contacted all members via email and provided study-related materials, including study aims and procedures; interested patients then contacted the study project members direct. Participants were screened and those eligible were scheduled for a telephone interview. Inclusion criteria included the following: male and female patients with self-reported PLD or GLD, aged ≥ 12 years, willing to participate in a 60- to 90-min telephone interview, willing to provide verbal informed consent (aged ≥ 18 years) or assent (aged 12–17 years), and able to speak English. Adult participants were provided $100 and adolescent participants and their caregivers were each provided $50 for the interviews.

### Qualitative interviews

After consent was obtained, in-depth interviews were conducted. Each interview was conducted by two experienced interviewers (SAM, OO-A) following a semi-structured interview guide. One served as the primary interviewer while the other took notes and monitored the need for additional questions or probes. Interviewers alternated roles across the interviews. All interviews were audio recorded and transcribed for data analysis.

**Fig. 1:**
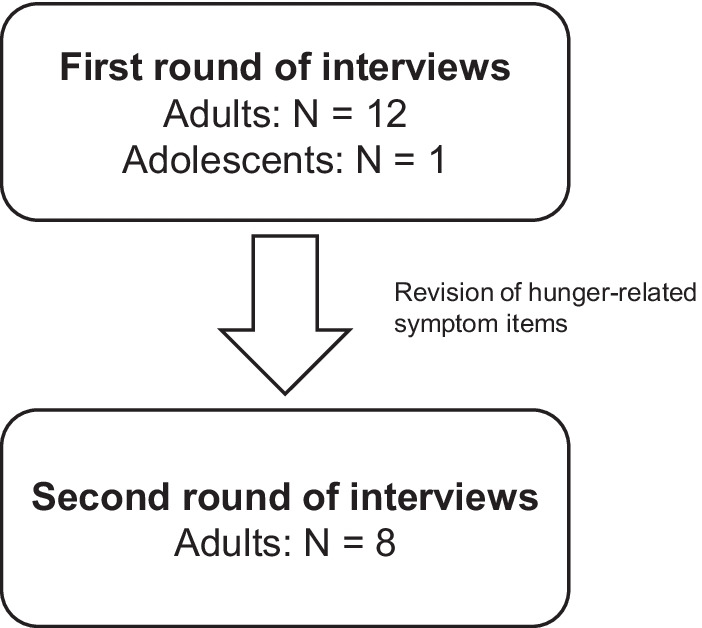
Design of interview process

During the first part of each interview, interviewers conducted a brief concept elicitation discussion by asking open-ended questions to obtain information on symptom concepts directly from the participants. Participants were then asked to provide their most bothersome symptoms. After the concept elicitation discussion, the interviewer asked the participants questions focused on their experiences of hunger-related symptoms and any related effects of these symptom experiences. Lastly, the interviewers conducted cognitive debriefing of the newly developed hunger-related items in all participants. After the first round of interviews (N = 13) were completed, revisions were made to the hunger-related symptom items; the revised hunger-related symptom items were then evaluated in the second round of interviews (N = 8; Fig. 1). Specifically, participants were asked to complete the newly developed items using a “think aloud” process [14], and interviewers probed further to understand how participants interpreted and selected an answer for each item. Additionally, participants evaluated both numeric rating and verbal response option in the newly developed questionnaire. Cognitive debriefing of the body temperature PRO measure and global items intended to assist with developing a responder threshold for evaluating clinically important changes was also conducted during the second round of interviews (N = 8).

### Analysis

Analyses followed standard qualitative data collection and analysis methods that included two main guiding principles: researcher neutrality and systematic process. Data were systematically collected in the form of field notes and audio files which were transcribed. Descriptive analyses were used to summarize the demographic and clinical data provided by participants at screening, and qualitative analysis of the interview data. The descriptive analyses included the computation of frequencies and percentages for categorical variables (e.g., gender, education) and the computation of means, standard deviations, and ranges for continuous variable (e.g., current age).

Immediately after each interview, RTI Health Solutions began the analysis process: research staff debriefed and recorded their initial thoughts and key points from the interview, using field notes. This step was followed by formal analysis of the field notes and the interview transcripts facilitated by Microsoft Excel (Microsoft Corporation; Redmond, Washington). Using the field notes and interview transcripts, dominant trends were identified in each interview and compared across the results of the other interviews [15] to generate themes or patterns in the way participants described their experiences with lipodystrophy, specifically hunger-related concepts. Saturation was assessed by comparing the results of the last five interviews with the results from the prior interviews to identify whether any new concepts emerged in the final interviews (thus indicating that additional interviews may be required to achieve saturation).

## Results

The participants’ characteristics are provided in Table 1. Interviews were conducted with one adolescent and 20 adults across the USA. In accordance with the known prevalence of PLD in females and the PLD predominance in this study, the majority of participants were female (95.2%); participants had an average age of 42.5 years (range 16–71 years). Minority groups comprised 23.8% of the sample: three participants were African American (14.2%), one was Asian Pacific Islander (4.8%), and one was Euro Asian (4.8%). Of the 21 participants interviewed, 17 had PLD (16 with FPL and one with acquired PLD [APL]), while only four participants had GLD. Of these four, three were African American; all reported current use of metreleptin for the treatment of their lipodystrophy. The mean body mass index for participants was 28.3 kg/m^2^ (range 13.3–46.6 kg/m^2^). Saturation was deemed to be achieved based on the comparison of the concepts reported during the last five interviews compared with the prior interviews.

**Table 1:** Demographic and clinical characteristics

Characteristics	Round 1 (N = 13)	Round 2 (N = 8)	Total (N = 21)
*Demographics*			
Age, years			
Mean	46.7	35.8	42.5
Range	16–71	19–58	16–71
Sex, n (%)			
Male	1 (7.7)	0 (0)	1 (4.8)
Female	12 (92.3)	8 (100)	20 (95.2)
Race/ethnicity, n (%)			
White	12 (92.3)	4 (50)	16 (76.2)
African American	0 (0)	3 (37.5)	3 (14.2)
Asian Pacific Islander	0 (0)	1 (12.5)	1 (4.8)
Euro Asian	1 (7.7)	0 (0)	1 (4.8)
Education, n (%)			
High school grade^a^	1 (7.7)	0 (0)	1 (4.8)
High school diploma or equivalent	0 (0)	1 (12.5)	1 (4.8)
Some college	4 (30.8)	5 (62.5)	9 (42.8)
College degree	5 (38.4)	1 (12.5)	6 (28.6)
Professional or advanced degree	3 (23.1)	1 (12.5)	4 (19.0)
Employment status, n (%)			
Full-time	2 (15.4)	1 (12.5)	3 (14.3)
Part-time	4 (30.8)	1 (12.5)	5 (23.8)
Not employed/retired	6 (46.1)	6 (75.0)	12 (57.1)
N/A^b^	1 (7.7)	0 (0)	1 (4.8)
Geographic location of participants			
Northeast	3	0	3 (14.3)
Northwest	2	1	3 (14.3)
South	1	1	2 (9.5)
Midwest	5	3	8 (38.1)
West	0	1	1 (4.8)
Mid-Atlantic	2	2	4 (19.0)
*Disease*			
Form of lipodystrophy, n (%)			
FPL	13 (100)	3 (37.5)	16 (76.2)
CGL	0 (0)	4 (50.0)	4 (19.0)
APL	0 (0)	1 (12.5)	1 (4.8)
BMI, kg/m^2^			
Mean	29.6	26.1	28.3
Range	24.6–46.6	13.3–45.9	13.3–46.6
Lipodystrophy treatment, n (%)			
Leptin	0 (0)	4 (50)	4 (19)

*APL* acquired partial lipodystrophy, *BMI* body mass index, *CGL* congenital generalized lipodystrophy, *FPL* familial partial lipodystrophy

^a^Adolescent, currently in grade school

^b^Adolescent, not employed

### Symptom and treatment effects: results of open-ended elicitation

Participant descriptions of their experiences were similar for those with PLD and GLD; thus, the results are provided together.

### Description of condition

Overall, participants reported rarely using the term “lipodystrophy” when describing their condition to others since most individuals not in healthcare are not familiar with it. Instead, they described their condition as a genetic disease where fat is improperly stored in the body. Specifically, participants provided examples such as “lack of fat cells,” “storage of fat in organs,” or “muscular body.”

### Lipodystrophy-related symptoms

**Fig. 2:**
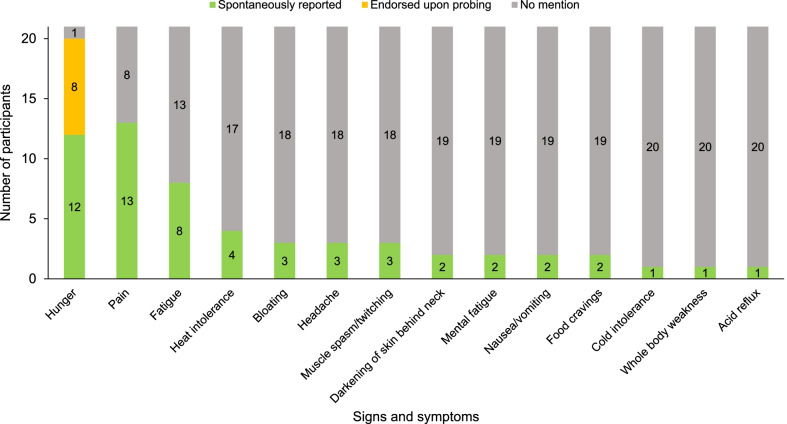
Symptoms and signs of lipodystrophy reported by study participants

The symptoms that participants reported were associated with their lipodystrophy, excluding comorbidities or complications (e.g., diabetes, as this was not the focus of the interviews), are summarized in Fig. 2 and Additional file 1: Table S1. Participants consistently reported two symptoms: increased hunger and pain. Hunger was either reported spontaneously (N = 12) or endorsed upon probing (N = 8) by all but one participant. One participant reported not experiencing increased hunger because of gastroparesis. This participant also reported a diagnosis of diabetes mellitus, though the duration of the diabetes or whether the gastroparesis was a complication of diabetes was not discussed. Pain was the most commonly reported spontaneous symptom (N = 13). The next most frequently reported spontaneous symptom after pain and hunger was fatigue (N = 8), followed by heat intolerance (N = 4).

### Most bothersome symptoms of lipodystrophy

**Fig. 3:**
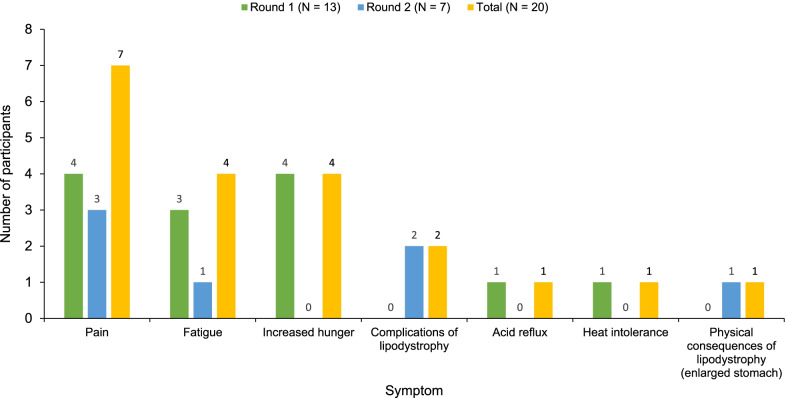
Most bothersome symptom of lipodystrophy

Of the 21 participants, 20 provided input on their most bothersome lipodystrophy-related symptom (Fig. 3 and Additional file 1: Table S2). Across both interview rounds, participants most frequently reported pain (N = 7) as the most bothersome symptom. Increased hunger and fatigue were ranked the second most bothersome symptoms, mentioned by four participants each. Illustrative quotes for the three most commonly reported bothersome symptoms of lipodystrophy are provided below.

#### Pain

“Yeah. It is like a muscle pain too. There are…it is also a muscle pain. There are certain days you can definitely tell the difference. Just in forms of functioning…But the body pain…it’s something…and I go and see a pain management specialist. I’m on pain medication, and it doesn’t help for a long time.” [FPL].

“I would say probably, of the things that I constantly experience, the nerve pain, especially in my hands, because I have it all over my body, but especially in my hands. It affects everything that you’re trying to do. I try to turn a key in a door, and I’ll get shooting nerve pain up my arm because it’s pressed on a nerve in my hand when I was trying to turn it. Things like that. I can’t open containers because of the nerve pain in my hands.” [FPL].

“The bone pain for sure. I mean I guess just because I don’t have any fat in those areas that all the pressure, any pressure is on them and, you know, walking and stuff like that, while I do it, I just, I kind of pay for it later. So, it’s just, you know, just aching, agonizing, just always there.” [FPL].

“The constant muscle pain because it makes it where I can’t function like a normal human being. I used to constantly hike, and I used to walk around, and I’d be able to do housework and everything else on a regular normal basis and not have to stop, and now I have to limit everything I do because of how bad I hurt.” [FPL].

“I guess like the leg pain and the arm pain. They always feel like they’re asleep. They have that tingly, prickly feeling. Well, it cuts down my activity level, like how much I can do in a day.” [FPL].

“Probably the pain. Just because I can’t do what I used to do. Like if I would want to go clean my whole house or a couple rooms, I have to do a little at a time. You just sort of have to do what your body will let you do, and then rest, and then you can continue doing it in a little while.” [APL].

#### Increased hunger

“I would say probably the wanting to eat. It literally affects me the entire time I’m awake. I mean, there’s never a minute I’m not thinking about food, and that affects my relationships. It affects pretty much everything I do.” [FPL].

“The eating, because… I mean, eating is part of your social life too. We don’t really go out to eat with other people much.” [FPL].

“I hate the hyperphagia…. But the problem is, is when I, when I start eating carbs, I can’t stop and the wallpaper looks good.” [FPL].

#### Fatigue

“I think for me it would be the fatigue. Because like I say, if I have had an active day or an active couple of days, then I’m kind of done for, for maybe 3 or 4 days… And I just feel like I’m not able to experience some of those life moments with as much joy or pleasure as what I should because the fatigue or the pain is kind of putting a damper on that.” [FPL].

“The chronic fatigue. Because that means I can’t get anything done during the day that needs to get done.” [FPL].

“But I’d say the most important one is the fatigue, because yesterday I went for an interview and I probably was outside for maybe… 3 or 4 h, and I was like unbelievably tired.” [CGL].

### Hunger-related symptoms

The report of increased hunger associated with lipodystrophy varied between participants. Specifically, one participant with FPL reported that she had never experienced increased hunger or other related symptoms, while nine participants stated that they had experienced these hunger-related symptoms in the past but no longer considered this an issue (three of these participants attributed this change to receiving leptin). Table 2 summarizes all the hunger-related symptoms provided by participants, including previous experiences. The most frequently reported concepts included increased hunger/appetite (N = 11) and an inability to satisfy hunger (N = 9). Participant descriptions of their hunger-related symptoms focused primarily on an increased appetite leading to overeating. An inability to feel full, which some participants described as a mental construct rather than a physical one, and cravings were also described. Examples of participants’ descriptions are:

“Starving, famished. I’m going to die if I don’t eat soon. If I didn’t control myself, I could put on a feedbag and eat all day long.” [FPL].

“I want to eat all the time, but usually my brain tells me to shup up. But yes, I would love to eat constantly, yes, but I can’t. I also had the gastric sleeve a couple of years ago, but it really didn’t help either.” [FPL].

“So it’s like weird, because it’s a couple hours. Like whatever I eat satisfies me for a little bit, but then I’m back to being hungry again. So then, like I would eat, and I would feel full, and then within like a half hour or 45 min, I would feel hunger.” [FPL].

**Table 2:** Hunger-related concepts reported by study participants

Symptoms	Participant/interview number																					Total
	1	2	3	4	5	6	7	8	9	10	11	12	13	14	15	16	17	18	19	20	21	
	FPL	FPL	FPL	FPL	FPL	FPL	FPL	FPL	FPL	FPL	FPL	FPL	FPL	FPL	CGL	CGL	FPL	APL	FPL	CGL	CGL	
Increased appetite/hunger (leading to excessive eating/eat more than others)	✓	✓	–	–	–	–	–	–	–	–	✓	✓	–	✓	✓	✓	✓	✓	–	✓	✓	11
Not feeling satisfied/full	–	–	✓	✓	–	–	–	–	–	–	–	✓	–	–	–	✓	✓	✓	✓	✓	✓	9
Food craving (most often carbs/sweets)	–	✓	–	✓	–	–	✓	–	–	–	–	✓	–	–	–	✓	✓	–	–	–	–	6

*APL* acquired partial lipodystrophy, *CGL* congenital generalized lipodystrophy, *FPL* familial partial lipodystrophy

✓Concept spontaneously reported by participant

“–” No mention of concept

The timing of the hunger-related symptoms also varied (Table 3). Seven participants reported feeling most hungry during evening hours, while five participants reported feeling most hungry upon waking up in the morning. While most participants reported experiencing hunger on a daily basis, four reported infrequent surges of hunger. In general, participants rated their experience with hunger as moderate to severe.

“I would rate it moderate. I think because I have figured out how to deal with it and, like I said, I’m trying to figure out whether objectively… But I think it’s moderate because it’s not totally overwhelming.” [FPL].

“Oh, definitely severe. Sometimes when I would take my blood sugar because I was diagnosed with diabetes at age 12 or 13, my blood sugar sometimes when I checked it, it wouldn’t even read it. It would just say high, because that’s how high my blood sugars were. The highest A1C I ever got up to was like 12. It was ridiculous.” [CGL].

**Table 3:** Timing of hunger

Symptoms	Participant/interview number																					Total
	1	2	3	4	5	6	7	8	9	10	11	12	13	14	15	16	17	18	19	20	21	
	FPL	FPL	FPL	FPL	FPL	FPL	FPL	FPL	FPL	FPL	FPL	FPL	FPL	FPL	CGL	CGL	FPL	APL	FPL	CGL	CGL	
Night eating/hungry at night	✓	–	–	–	–	✓	–	–	✓	✓	✓	–	–	–	–	✓	–	–	–	–	✓	7
Hungry in morning upon waking up/few hours upon waking	✓	–	✓	–	–	–	–	–	–	–	–	–	✓	–	–	✓	✓	–	–	–	–	5
Surge of hunger (in frequent unusual hunger)/hunger fluctuates	–	–	–	–	✓	✓	✓	–	–	–	–	–	–	–	–	–	–	✓	–	–	–	4
Constant thoughts about food	–	–	✓	–	–	–	–	–	–	–	–	✓	–	–	✓	–	–	–	–	–	–	3

*APL* acquired partial lipodystrophy, *CGL* congenital generalized lipodystrophy, *FPL* familial partial lipodystrophy

✓Concept spontaneously reported by participant

“–” No mention of concept

#### Pain

Because the focus of the interviews was on the hunger-related symptoms, an in-depth exploration of pain was not included in the interviews. However, pain was spontaneously reported by 13 participants, as summarized in Table 4.

**Table 4:** Spontaneous reports of pain as a symptom of lipodystrophy

Participant ID	1	2	3	4	5	6	7	8	9	10	11	12	13	14	15	16	17	18	19	20	21	
Lipodystrophy subtype	FPL	FPL	FPL	FPL	FPL	FPL	FPL	FPL	FPL	FPL	FPL	FPL	FPL	FPL	CGL	CGL	FPL	APL	FPL	CGL	CGL	
General body pain^a^		✓		✓	✓	✓				✓							✓		✓			N = 7
Muscle pain	✓			✓						✓				✓				✓				N = 5
Bone/joint pain					✓		✓			✓						✓					✓	N = 5
Neuropathy							✓										✓	✓				N = 3
Headache/migraine							✓															N = 1

*APL* acquired partial lipodystrophy, *CGL* congenital generalized lipodystrophy, *FPL* familial partial lipodystrophy

✓Concept spontaneously reported by participant

^a^Often described as related to lack of subcutaneous fat

While the type and description of the pain varied across all participants, many participants reported more than one type of pain; they attributed their pain to a lack of fat and therefore an inability to cushion or protect certain areas of the body, such as the feet and buttocks. General body pain or pain in the limbs was most frequently reported (N = 7), followed by muscle pain (N = 5), bone/joint pain (N = 5), nerve pain (N = 3), and headache/migraine (N = 1). Example quotes from participants for each type of pain reported are:

“Without a doubt, losing the fat on your body is an unbelievable amount of body pain. The bottom of my feet, it feels like I’m walking on…I can just remember walking on the beach as a kid and the sand felt really nice. Now if I walk on the beach, it feels like I’m stepping on glass. Like when I walk, it can really hurt. It feels like I have no cushion on my feet, and the same thing with my joints.” [FPL].

“It is like a muscle pain too. There are…it is also a muscle pain. There are certain days you can definitely tell the difference.” [FPL].

“I also have like a lot of like pains. Like it feels like they’re on the inside of my bones and not necessarily like just regular pain.” [CGL].

“Yeah. I have it [nerve pain]. It’s mostly in my feet. It’s like numbness, loss of feeling in some parts of my feet. Also, nerve pain in the feet, but I have nerve pain, like all over my body. I mean my hands, my elbows.” [FPL].

“I mean, migraines are up there when they hit, but it’s not constant.” [FPL].

### Impact of hunger-related symptoms

**Fig. 4:**
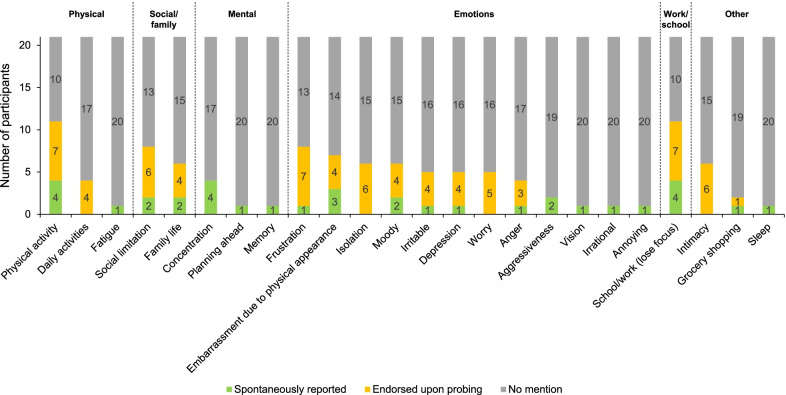
Impacts of hunger-related symptoms reported by study participants

Interview participants were asked to describe how their hunger-related symptoms affected their lives. Figure 4 and Additional file 1: Table S3 summarize both the impacts that were reported spontaneously and those that were endorsed upon probing. Frequently reported impacts were limitations in physical and social activities, which were reported by 11 and eight participants, respectively. Additionally, participants frequently reported impacts on their emotions, which included frustration (N = 8), embarrassment (N = 7), isolation (N = 6), and altered mood (N = 6).

### The most difficult aspect of having lipodystrophy

At the end of the interview, participants were asked to describe the hardest part of having lipodystrophy. Of the 15 participants who provided feedback, hunger-related issues were most frequently reported by participants (N = 6). Specifically, these were inability to satisfy their hunger (N = 3), lack of control over quantity of food consumed (N = 1), distraction as result of hunger (N = 1), and food cravings (N = 1). One participant reported bone and muscle pain as the most difficult aspect of having lipodystrophy.

Additional aspects mentioned included emotional impacts (N = 2), as well as social impacts, sleep, physical limitation, weight gain, and route of medicine administration, which were each reported by one participant.

### Cognitive debriefing interview

The cognitive debriefing portion of the interviews was initiated after participants had described their experiences with symptoms and signs. Minor modifications to the hunger-related items were made following both rounds of interviews, including selecting the items that were the most relevant and easiest to complete as reported by participants. These modifications are detailed in Additional file 1: Table S4. Importantly, the developed hunger-related symptom items were correctly interpreted and easily completed by the participants. Participant reports also supported that the items were relevant and reflected their experience with these symptoms due to lipodystrophy. Similarly, the temperature and global impression of change and severity items that were tested in round 2 were found to be easy to understand and complete.

## Discussion

The main objective of this research was to understand the experiences of hunger-related symptoms of patients with lipodystrophy, and to evaluate the content validity of items developed to assess this concept. All but one participant endorsed having issues with hunger-related symptoms at some point in their condition (even if not experiencing currently); therefore, an in-depth description of the symptoms and impact of these symptoms was achieved. Participants most often reported symptoms of an increased appetite and not feeling full. In addition, all participants were able to provide input into each of the items during the cognitive debriefing. Specifically, the hunger-related symptom items were correctly interpreted and easily completed by the participants. Participant reports also supported that the items were relevant and reflected their experience with hunger-related symptoms due to lipodystrophy.

The results of the individual interviews indicated that hunger-related symptoms were considered a current issue for just over half (N = 11) of the participants. Therefore, those conducting clinical research may consider whether the clinical trial inclusion criteria should include a requirement of experiencing hunger-related symptoms at study enrollment or if analyses of changes in these symptoms should be limited to those experiencing these symptoms at baseline. It will be important to consider these patient-centric issues in the evaluation of treatments for lipodystrophy by regulators and payors.

Our findings are in agreement with a qualitative analysis by Arpana et al. that also indicated a significant number of patient-centered impacts associated with lipodystrophy, including significant hunger, pain, fatigue, depression, altered physical appearance, and anxiety [16]. While not a focus of the present study, our findings demonstrated that emotion/affect were often called out by patients with lipodystrophy. This result is consistent with a previous study where 16 patients with lipodystrophy were studied alongside 20 females with breast cancer and 20 obese patients [17]. Mood and anxiety disorders were observed at a high rate in the lipodystrophy patients. In addition, the lipodystrophy subgroup was found to have higher scores on instruments designed to measure the interference of pain with daily living (sub-score on the Westhaven-Yale multidimensional pain inventory [WHYMPI]), as well as the paranoid subscale in the Symptom Checklist-90-Revised (SCL-90-R) and the cues that trigger food craving sub-scale in the food cravings questionnaire-trait (FCQ-T) scale. In our study, patients did not spontaneously report paranoid behavior but they did report often feeling isolated and embarrassed by their physical appearance.

One limitation of our analysis is that fewer patients with GLD (N = 4) were recruited than planned. However, the ratio of GLD to PLD patients in this study is similar to the reported relative prevalence of each disease in the general population [18]. While differences in the hunger-related symptoms reported for participants with PLD versus GLD were not observed, more interviews in patients with GLD should be conducted to confirm these results. Another limitation of the study is that the subjective patient interviews were not performed with objective measures of the severity of lipodystrophy, such as imaging and laboratory assessments. For example, it would be interesting to see whether there is a correlation between patient-reported hunger and leptin levels. Leptin is known to suppress hunger, which is an explanation for the profound hyperphagia observed in states of leptin deficiency, including lipodystrophy [3]. In addition, while patients were asked to provide the symptoms and impacts that they believe are related to lipodystrophy, it is possible that some of the reported concepts may be due to other comorbid conditions.

Finally, while not a focus of the interviews, the concept elicitation results demonstrated that pain was a frequently reported and bothersome symptom in this patient population. Pain is an extremely complex sensation that can be modulated by factors within the tissue, along sensory nerves, in the spinal cord, and in various other central nervous system sites. The location and type of pain described by participants varied. Many participants attributed this pain to the lack of subcutaneous fat, which could provide a mechanical explanation for the pain. However, it should be noted that leptin receptors are expressed in many central nervous system nuclei beyond the hypothalamus [19], and could account, at least in part, for modulation of pain. Therefore, those conducting clinical research studies may want to explore this concept further with additional qualitative research, and include a pain assessment in upcoming clinical trials if it is anticipated that this symptom would improve with treatment.

## Conclusions

In summary, this qualitative research provided evidence to support the use of the hunger-related symptom items in clinical trials. Recommended next steps are to conduct qualitative interviews with more participants with GLD, and to evaluate the psychometric properties of the hunger-related symptom items in clinical trials.

## Supplementary Information

**Additional file 1. Table 1**. Symptoms and signs reported by study participants. **Table 2**. Most bothersome symptom ranking. **Table 3**. Impact of hungerrelated symptoms reported by study participants. **Table 4**. Item-tracking matrix.

## Data Availability

Not applicable.

## Abbreviations

APL: Acquired partial lipodystrophy
CGL: Congenital generalized lipodystrophy
FPL: Familial partial lipodystrophy
GLD: Generalized lipodystrophy
PLD: Partial lipodystrophy
PRO: Patient-reported outcome
