# Supplementary material for: Qualitative interviews in patients with lipodystrophy to assess the patient experience: evaluation of hunger and other symptoms
Source: J Patient Rep Outcomes. 2022 Jul 29;6:84. doi: 10.1186/s41687-022-00486-3 (PMC9338178; doi:10.1186/s41687-022-00486-3)
Supplement: Supplementary file 1 — Additional file 1. Table 1. Symptoms and signs reported by study participants. Table 2. Most bothersome symptom ranking. Table 3. Impact of hungerrelated symptoms reported by study participants. Table 4. Item-tracking matrix. [file 41687_2022_486_MOESM1_ESM.docx]

Table S1. Symptoms and signs reported by study participants

| Symptoms | Concept elicitation participants | | | | | | | | | | | | | | | | | | | | | |
| --- | --- | --- | --- | --- | --- | --- | --- | --- | --- | --- | --- | --- | --- | --- | --- | --- | --- | --- | --- | --- | --- | --- |
|  | **1** | **2** | **3** | **4** | **5** | **6** | **7** | **8** | **9** | **10** | **11** | **12** | **13** | **14** | **15** | **16** | **17** | **18** | **19** | **20** | **21** | **Total** |
|  | **FPL** | **FPL** | **FPL** | **FPL** | **FPL** | **FPL** | **FPL** | **FPL** | **FPL** | **FPL** | **FPL** | **FPL** | **FPL** | **FPL** | **CGL** | **CGL** | **FPL** | **APL** | **FPL** | **CGL** | **CGL** |  |
| Hunger | S | S | S | P | S | S | P | – | P | P | P | S | S | P | S | S | S | S | P | S | P | S = 12 P = 8 |
| Pain | S | S | – | S | S | S | S | – | – | S | – | – | – | S | – | S | S | S | S | – | S | S = 13 |
| Fatigue | – | S | S | – | – | S | S | – | – | – | S | – | – | S | – | S | S | – | – | – | – | S = 8 |
| Heat intolerance | S | – | S | S | – | – | – | – | – | – | – | – | S | – | – | – | – | – | – | – | – | S = 4 |
| Bloating | – | – | – | – | – | – | S | – | – | – | S | S | – | – | – | – | – | – | – | – | – | S = 3 |
| Headache | – | S | – | – | – | – | S | – | – | – | – | – | – | – | – | – | – | – | – | – | S | S = 3 |
| Muscle spasm/twitching | – | – | – | – | – | S | – | – | – | – | – | – | – | S | – | S | – | – | – | – | – | S = 3 |
| Darkening of skin behind neck | S | – | – | – | – | – | – | – | – | – | – | – | – | – | – | S | – | – | – | – | – | S = 2 |
| Mental fatigue | – | S | – | – | – | – | – | – | – | – | – | – | – | – | – | – | S | – | – | – | – | S = 2 |
| Nausea/vomiting | – | – | – | – | – | – | – | S | S | – | – | – | – | – | – | – | – | – | – | – | – | S = 2 |
| Food cravings | – | – | – | S | – | – | – | – | – | – | – | – | – | – | – | S | – | – | – | – | – | S = 2 |
| Cold intolerance | – | – | S | – | – | – | – | – | – | – | – | – | – | – | – | – | – | – | – | – | – | S = 1 |
| Whole body weakness | – | – | – | – | – | – | – | – | – | – | – | – | – | – | – | – | – | S | – | – | – | S = 1 |
| Acid reflux | – | – | – | – | – | – | – | S | – | – | – | – | – | – | – | – | – | – | – | – | – | S = 1 |

Abbreviations: APL = acquired partial lipodystrophy; CGL = congenital generalized lipodystrophy; FPL = familial partial lipodystrophy.

S indicates a symptom that participants spontaneously reported.

P indicates a symptom that participants endorsed upon probing.

“–“ no mention of concept.

Table S2. Most bothersome symptom ranking

| **Symptom** | **Round 1 (*N* = 13)** | **Round 2 (*N* = 7)** | **Total (*N* = 20)** |
| --- | --- | --- | --- |
| Most bothersome |  |  |  |
| Pain | 4 | 3 | 7 |
| Fatigue | 3 | 1 | 4 |
| Increased hunger | 4 | 0 | 4 |
| Complications of lipodystrophy | 0 | 2 | 2 |
| Acid reflux | 1 | 0 | 1 |
| Heat intolerance | 1 | 0 | 1 |
| Physical consequence of lipodystrophy (enlarged stomach) | 0 | 1 | 1 |

Table S3. Impact of hunger-related symptoms reported by study participants

|  | **Concept elicitation participants** | | | | | | | | | | | | | | | | | | | | |  |
| --- | --- | --- | --- | --- | --- | --- | --- | --- | --- | --- | --- | --- | --- | --- | --- | --- | --- | --- | --- | --- | --- | --- |
|  | **1** | **2** | **3** | **4** | **5** | **6** | **7** | **8** | **9** | **10** | **11** | **12** | **13** | **14** | **15** | **16** | **17** | **18** | **19** | **20** | **21** |  |
|  | **FPL** | **FPL** | **FPL** | **FPL** | **FPL** | **FPL** | **FPL** | **FPL** | **FPL** | **FPL** | **FPL** | **FPL** | **FPL** | **FPL** | **CGL** | **CGL** | **FPL** | **APL** | **FPL** | **CGL** | **CGL** | **Total** |
| **Physical** | | | | | | | | | | | | | | | | | | | | | | |
| Physical activity | – | P | S | – | P | – | – | P | – | – | P | S | P | – | – | P | P | S | – | S | – | S = 4 P = 7 |
| Daily activities | – | – | P | – | – | – | – | – | – | – | – | P | – | – | – | P | P | – | – | – | – | P = 4 |
| Fatigue | – | – | – | – | – | – | – | – | – | – | – | – | – | – | – | – | – | S | – | – | – | S = 1 |
| **Social/family** | | | | | | | | | | | | | | | | | | | | | | |
| Social limitation | P | – | P | P | P | P | – | – | – | – | – | P | – | – | – | S | S | – | – | – | – | S = 2 P = 6 |
| Family life | – | – | S | – | P | P | – | – | – | – | – | P | – | – | – | S | P | – | – | – | – | S = 2 P = 4 |
| **Mental** | | | | | | | | | | | | | | | | | | | | | | |
| Concentration | S | – | S | – | – | – | – | – | – | – | – | – | – | – | – | S | – | – | – | S | – | S = 4 |
| Planning ahead | – | – | – | – | – | – | – | – | – | – | – | – | – | – | – | – | – | S | – | – | – | S = 1 |
| Memory | – | – | – | – | – | – | – | – | – | – | – | – | – | – | – | S | – | – | – | – | – | S = 1 |
| **Emotions** | | | | | | | | | | | | | | | | | | | | | | |
| Frustration | P | P | P | P | P | – | – | – | – | – | – | S | – | – | – | – | P | P | – | – | – | S = 1 P = 7 |
| Embarrassment due to physical appearance | – | S | P | S | P | P | – | – | – | – | – | S | – | – | – | – | P | – | – | – | – | S = 3 P = 4 |
| Isolation | – | P | P | – | P | P | – | – | – | – | – | – | – | – | – | – | P | P | – | – | – | P = 6 |
| Moody | P | – | P | P | – | P | – | – | – | – | – | – | – | – | – | S | S | – | – | – | – | S = 2 P = 4 |
| Irritable | P | – | – | P | – | P | – | – | P | – | – | – | – | – | – | – | S | – | – | – | – | S = 1 P = 4 |
| Depression | – | P | S | – | – | P | – | – | – | – | – | – | – | – | – | – | P | P | – | – | – | S = 1 P = 4 |
| Worry | – | P | – | – | P | P | – | – | – | – | – | – | – | – | – | – | P | P | – | – | – | P = 5 |
| Anger | – | – | – | P | P | P | – | – | – | – | – | S | – | – | – | – | – | – | – | – | – | S = 1 P = 3 |
| Anxiety | – | – | – | – | – | – | – | – | – | – | – | – | – | – | – | – | S | S | – | – | – | S = 2 |
| Aggressiveness | – | – | – | – | – | – | – | – | – | – | – | – | – | – | – | S | – | – | – | – | - | S = 1 |
| Vision | – | – | – | – | – | – | – | – | – | – | – | – | – | – | – | S | – | – | – | – | – | S = 1 |
| Irrational | – | – | – | – | – | – | – | – | – | – | – | – | – | – | – | – | S | – | – | – | – | S = 1 |
| Annoying | – | – | – | – | – | – | – | – | – | – | S | – | – | – | – | – | – | – | – | – | – | S = 1 |
| **Work/school** | | | | | | | | | | | | | | | | | | | | | | |
| School/work (lose focus) | S | – | P | P | P | – | – | P | – | – | – | P | P | – | – | S | S | P | – | – | S | S = 4 P = 7 |
| **Other** | | | | | | | | | | | | | | | | | | | | | | |
| Intimacy | – | P | P | P | – | – | – | P | – | – | – | – | – | – | – | – | P | P | – | – | – | P = 6 |
| Grocery shopping | – | – | – | – | – | S | – | – | – | – | P | – | – | – | – | – | – | – | – | – | – | S = 1 P = 1 |
| Sleep | – | – | – | – | – | – | – | – | – | S | – | – | – | – | – | – | – | – | – | – | – | S = 1 |

Abbreviations: APL = acquired partial lipodystrophy; CGL = congenital generalized lipodystrophy; FPL = familial partial lipodystrophy.

S indicates an impact that participants spontaneously reported.

P indicates an impact that participants endorsed upon probing.

“–“ no mention of concept.

Table S4. Item-tracking matrix

| **Round 1  (*N* = 13)** | **Round 1 interview findings and resulting actions** | **Round 2  (*N* = 8)** | **Round 2 interview findings and resulting actions** | **Final** |
| --- | --- | --- | --- | --- |
| **Instructions** | | | | |
| *For each of the following questions, please choose the one answer that best describes your experiences during the past 24 hours.* | All participants understood the instructions and the recall period of 24 hours.  **Retained without modification** | *For each of the following questions, please choose the one answer that best describes your experiences during the past 24 hours.* | All participants understood the instructions and the recall period of 24 hours.  **Retained without modification** | *For each of the following questions, please choose the one answer that best describes your experiences during the past 24 hours.* |
| **Lipodystrophy hunger diary** | | | | |
| 1. *Overall, how would you rate your hunger today?*   **Response options** NRS from 0 (not hungry at all) to 10 (hungriest possible) | See item No. 2 below  **Omitted** |  |  |  |
| 1. *Overall, how would you rate your hunger today?*   **Response options** NRS from 0 (never felt hungry) to 10 (extremely hungry all day) | Participants understood and interpreted the item consistently.  10 of the participants stated a preference for item 2 and the anchors tested with the NRS  **Item 2 and NRS were retained** | 1. *Overall, how would you rate your hunger today?*   **Response options** NRS from 0 (never felt hungry) to 10 (extremely hungry all day) | Participants understood and interpreted the item consistently; however, participants’ interpretation was similar to item No. 3. Additionally, the majority of participants across both rounds selected item No. 3 as the most relevant item.  **Omitted** |  |
| 1. *How would you rate your hunger at its worst today?*   **Response options** NRS from 0 (not hungry at all) to 10 (hungriest possible) | See item No. 4 below |  |  |  |
| 1. *How would you rate the highest level of hunger you felt today?*    Not hungry at all   A little hungry   Moderately hungry   Quite hungry   Extremely hungry | Participants understood and interpreted the item consistently.  11 participants stated a preference for item 4 and the VRS.  **Item 4 and VRS were retained** | 1. *How would you rate the highest level of hunger you felt today?*    Not hungry at all   A little hungry   Moderately hungry   Quite hungry   Extremely hungry | All participants understood and interpreted the item correctly.  Participants were easily able to select a response using the VRS.  **Retained without modification** | 1. *How would you rate the highest level of hunger you felt today?*    Not hungry at all   A little hungry   Moderately hungry   Quite hungry   Extremely hungry |
| 1. *How would rate your hunger at its least today?*   **Response options** NRS from 0 (not hungry at all) to 10 (hungriest possible) | See item No. 6 below |  |  |  |
| 1. *How would you rate the lowest level of hunger you felt today?*    Not hungry at all   A little hungry   Moderately hungry   Quite hungry   Extremely hungry | Participants understood and interpreted the item consistently.  10 of the participants stated a preference for item 6, and nine participants preferred the VRS evaluated with item 6  **Item 6 and VRS were retained** | 1. *How would you rate the lowest level of hunger you felt today?*    Not hungry at all   A little hungry   Moderately hungry   Quite hungry   Extremely hungry | All participants understood and interpreted the item correctly.  Participants were easily able to select a response using the VRS.  **Retained without modification** | 1. *How would you rate the lowest level of hunger you felt today?*    Not hungry at all   A little hungry   Moderately hungry   Quite hungry   Extremely hungry |
| 1. *How much of the time did you feel hungry today?*    None of the time   A little of the time (just before meals)   Some of the time (off and on throughout the day)   Most of the time (not after meals)   All of the time (even after meals) | Participants understood and interpreted the item consistently.  Participants were easily able to select a response using both VRS.  **Retained without modification** | 1. *How much of the time did you feel hungry today?*    None of the time   A little of the time (just before meals)   Some of the time (off and on throughout the day)   Most of the time (not after meals)   All of the time (even after meals) | All participants understood and interpreted the item correctly.  Participants were easily able to select a response using the VRS.  **Retained without modification** | 1. *How much of the time did you feel hungry today?*    None of the time   A little of the time (just before meals)   Some of the time (off and on throughout the day)   Most of the time (not after meals)   All of the time (even after meals) |
| 1. *How often did you feel full after eating meals or snacks today?*    Never   Once or twice   About half of the time   Most of the time   Every time | Participants understood and interpreted the item consistently.  Participants were easily able to select a response using both VRS.  **Retained without modification** | 1. *How often did you feel full after eating meals or snacks today?*    Never   Once or twice   About half of the time   Most of the time   Every time | All participants understood and interpreted the item correctly.  Participants were easily able to select a response using the VRS.  **Retained without modification** | 1. *How often did you feel full after eating meals or snacks today?*    Never   Once or twice   About half of the time   Most of the time   Every time |

Abbreviations: NRS = numeric rating scale; VRS = verbal rating scale.
